# Impact of diagnosis-intervention packet payment on the consistency of hospitalization expenses across different medical insurance schemes in China

**DOI:** 10.3389/fpubh.2025.1556234

**Published:** 2025-05-07

**Authors:** Jin Zi-Qian, Li Yi-Le, Tao Ying-Ying, Shen Ke-Yi, Lin Xin-Hao, Pei Tong, Li Cheng-Cheng, Wu Dan, Meng Xue-Hui

**Affiliations:** School of Humanities and Management, Zhejiang Chinese Medical University, Hangzhou, China

**Keywords:** Diagnosis-Intervention Packet (DIP), China, payment reform, health insurance schemes, expense consistency

## Abstract

**Background:**

The Diagnosis-Intervention Packet Payment (DIP) system is regarded as a localized cost-control strategy in China. It aims to improve healthcare efficiency, curb the growth of medical expenses, and optimize the allocation of medical resources among diverse groups.

**Objective:**

This study aims to assess the impact of DIP payment reforms on differential changes in patients’ hospitalization expense and to explore the degree of concentration of hospitalization expense for patients with different insurance schemes undergoing treatment for typical diseases, with a view to providing policy recommendations for improving the medical insurance system.

**Methods:**

Data were collected from patients with cerebral infarction (CI) and coronary atherosclerotic heart disease (CAD) treated at primary, secondary, and tertiary hospitals in S City of China, from 2020 to 2023. Patients were classified into the Urban Employees’ Basic Medical Insurance (UEBMI) group and the Urban and Rural Residents’ Medical Insurance (URRMI) group based on two health insurance schemes. Propensity Score Matching (PSM) was employed to ensure a balanced sample. The changes and trends in hospitalization expenses across different groups were analyzed using the interquartile Range (IQR), standard deviation (SD), and concentration index.

**Results:**

Post-DIP reform, hospitalization expenses for patients with different diseases at various levels of hospitals have decreased annually. Regarding expenses variation, the standard deviation (SD) of hospitalization expenses for both UEBMI and URRMI exhibited a downward trend, with a decrease in the double-difference value each year. From the perspective of expenses concentration, all concentration indices were less than 0 (statistically significant, *p* < 0.01), indicating a higher concentration in hospitalization expenses for UEBMI.

**Conclusion:**

The DIP reform can effectively increase the concentration of hospitalization expenses, reduce the variability of changes in hospitalization expenses for both UEBMI and URRMI, and drive medical practices toward standardization and consistency. However, the degree of this expense reduction varies among the hospitals at all levels.

## 1 Introduction

Medical insurance payments are an important lever for regulating medical service behaviors and guiding the allocation of medical resources (1). With economic development, demographic shifts, and changes in disease patterns, medical expenses have been rising globally. Many countries have implemented medical insurance payment reform to control expenses growth (2–4). Controlling medical expenses has also become a crucial task for the medical insurance sector in China and an essential part of deepening the reform of the medical and healthcare system. The traditional Chinese medical insurance payment method is fee-for-service, which often results in overutilization of medical services, excessive increases in medical expenses, and waste of healthcare resources (5). In response to escalating healthcare expenditures, China implemented Diagnosis-Related Group (DRG) payment reform as a cost-containment strategy, which demonstrated initial efficacy in standardizing medical expenditure patterns during the implementation phase. Studies have confirmed that DRG payment helps reduce disparities in medical expenses, suggesting that healthcare providers are more likely to follow clinical guidelines and provide effective treatment after assuming the risk of overspending (6, 7). However, over time, due to a variety of conditions, DRC has not been able to better realize its function in China (8–10). After continuous exploration, China launched a pilot program of the “Diagnosis-Intervention Package” (DIP) payment method based on its conditions (11). Rooted in China’s unique national conditions and grounded in clinical practice, DIP provides a bottom-up payment reform approach. Compared to the DRG system, DIP is simpler and less challenging to implement. It adopts a “coarse-to-fine” classification method to refine the categorization of medical cases. Additionally, the DIP system has lower medical record supervision and informatization requirements, making it more suitable for implementation in hospitals in second and third-tier cities (12). Therefore, DIP is considered a localized expense control method in China.

To achieve universal health coverage and equitable allocation of medical resources, the Chinese government established a unified urban and rural resident basic medical insurance system in 2016, New Rural Cooperative Medical Scheme (NRCMS) and Urban Residents’ Basic Medical Insurance Program (URBMI) were merged into the Urban and Rural Residents’ Basic Medical Insurance (13). As of the end of 2023, the number of people participating in Urban and Employee Basic Medical Insurance was 370,938,800 and the number of people participating in Urban and Rural Resident Basic Medical Insurance was 962,930,200. The operation of the basic medical insurance system is safe and stable. However, some researchers have pointed out that gaps still exist in healthcare utilization between UEBMI and URRMI (14). The main differences contributing to these disparities include target population, funding sources, funding levels, and administrative and benefit packages (15, 16). In addition to this, the health insurance payment method also affects the patients’ hospitalization expenses. Compared with the traditional fee-for-service (FFS) payment method, after the implementation of the DRG payment method reform, the patients’ hospitalization expenses have been significantly controlled, and the patients’ hospitalization burden has been reduced to a certain extent (17, 18). However, for primary healthcare institutions, due to gaps in medical quality, operational complexity, and the unpredictability of healthcare provider behavior, it is difficult to adapt to DRG reforms in a short period. Therefore, disparities in hospitalization expenses between different medical insurance schemes still exist. The DIP payment method explored by the Chinese government during the reform of the medical insurance system is considered to have similar effects to DRG payment (19). Studies have shown that the direct effect of the DIP reform is a significant reduction in the average hospitalization expenses per patient and the cost of medicines (20, 21), which can optimize the structure of medical expenses, effectively control cost growth, and inhibit waste of healthcare resources (22, 23). Compared to DRG-based payment models, DIP can allow for more homogeneous resource utilization within groups (24). For example, it was found that DIP led to a decrease in per-hospitalization expenses for both UEBMI and URRMI, while DRG payments led to an 11.2 percent increase in per-hospitalization expenses for URRMI (25).

However, due to the short implementation time of the DIP payment method in China, there is a lack of research on the impact of DIP reform on the hospitalization expenses of patients with different medical insurance schemes. Most of the studies have focused on changes in hospitalization expenses. In addition, studies have shown that health care provider behavior is a key factor in the variation in hospitalization expenses. Physicians’ diagnostic and treatment preferences affect hospitalization expenses more than the severity of a given illness (26). Some scholars have pointed out that after the DRG reform, doctors’ behavior is more in line with clinical norms, there is less variation in patients’ hospitalization expenses, and the cost changes are mainly in the cost of drugs and related materials (27–29). Previous studies by foreign scholars have also confirmed that DRG reforms can significantly affect physicians’ service behaviors, Huertaet et al. analyzed the differences in the efficiency of different healthcare organizations using data from the 2005 healthcare services survey conducted by the American Hospital Association and found that in hospitals that use the DRG payment model, the difference in expenditures is small, and the healthcare efficiency is negatively correlated with the expenses, indicating that there is a link between high costs and healthcare services are associated with low efficiency (30). Because of the similarities between the DIP payment method and the DRG payment method, they would have the same effect in regulating provider behavior and reducing hospitalization expenses variation. However, existing studies in China on the impact of Diagnosis-Intervention Packet (DIP) payment reform on inpatient hospitalization expenses have inadequately explored the consistency of expenditure patterns.

Therefore, to further supplement the effect of DIP reform on the consistency of patients’ hospitalization expenses, this study selects primary, secondary, and tertiary hospitals in a DIP pilot city in China as a research sample to analyze the differential changes in the hospitalization expenses of UEBMI and URRMI at all levels of hospitals in that city during the implementation of the DIP policy, and to provide policy recommendations for the improvement of the healthcare insurance system. This study aims to assess the impact of DIP payment reforms on differential changes in patients’ hospitalization expenses and to explore the degree of concentration of hospitalization expenses for patients with different insurance schemes undergoing treatment for typical diseases.

## 2 Methods

### 2.1 Data source

The City of S in China began implementing the DIP payment reform in January 2021. According to the survey, by the end of 2021, the DIP payment method reform in S City, China, had achieved full coverage in 10 counties (cities and districts), encompassing all primary, secondary, and tertiary hospitals and all inpatient diseases. S City took the lead in the province in completing the four full coverage of the integrated area, designated medical institutions, inpatient diseases, and the integrated fund. The data used in this study span from January 2020 to December 2023, encompassing variables such as patient gender, age, the level of medical institutions, disease code, out-of-pocket (OOP) cost, total medical expenses, and LOS. Due to the high number of cases and medical expenses of patients with cerebral infarction and coronary atherosclerotic heart disease, the study selected UEBMI and URRMI treated for these diseases at primary, secondary, and tertiary hospitals in S City from 2020 to 2023 to investigate the effect of DIP payment reform on the homogeneity of hospitalization costs for patients with different health insurance schemes.

### 2.2 Study design

**Figure 1:**
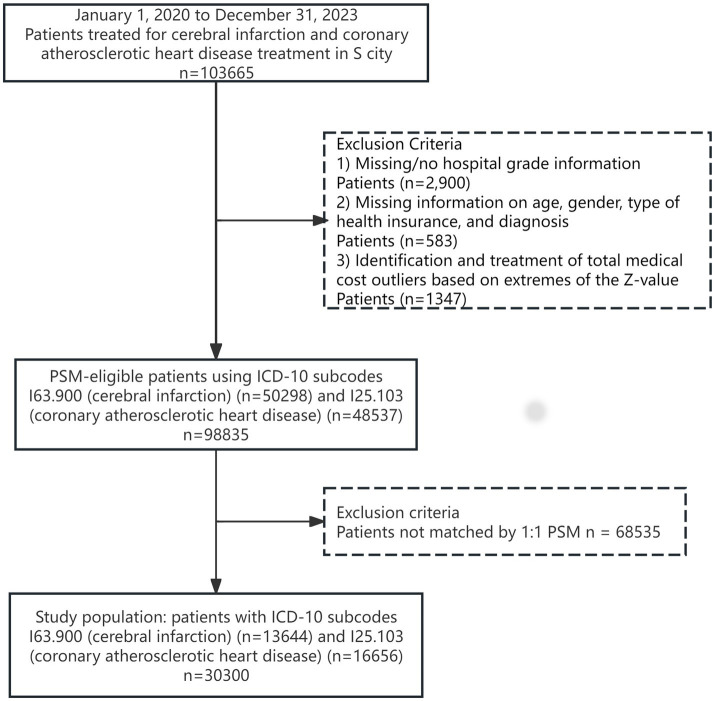
Schematic diagram of attrition in the study population.

During the preprocessing of the obtained 103,665 data records, this study used WPS 2021 software to delete missing values and SPSS 27 software to delete medical expense outliers based on the Z-score extreme value identification and processing method, retaining 98,835 data records—establishment of the URRMI group and the UEBMI group on this basis. Using age and gender as covariates, Stata 17.0 software was utilized to perform 1:1 Propensity Score Matching (PSM) for the URRMI and UEBMI groups year by year, with a matching caliper value of 0.01. The specific selection and exclusion process of the study population is shown in Figure 1.

### 2.3 Statistical analysis

This study aims to compare the disparities and changing trends in hospitalization expenses for treating typical conditions between the URRMI and UEBMI groups from 2020 to 2023, to assess the impact of DIP payment reform on the homogeneity of hospitalization expenses. The degree of variation in hospitalization expenses is expressed as standard deviation (SD), box plot joint interquartile range (IQR). Smaller values of standard deviation indicate a more concentrated distribution of hospitalization expenses. Similarly, the size of the interquartile range (IQR) can be reflected in a box plot to determine the degree of concentration. A smaller IQR value indicates a more concentrated expense distribution. In addition, the concentration index was chosen to reflect the distribution of hospitalization expenses across treatment modalities. Also, the Kruskal-Wallis test was used to compare the concentration index of each year and *p*-value was calculated. The formula for calculating the concentration index is as follows.


$$\mathrm{Concentration index}=\frac{2cov\left({h,r}_{i}\right)}{\bar{x\;}}$$

In this equation, cov (h, ri) denotes the covariance between the cost of hospitalization per patient (hi) and the rank of each patient in the sample (ri). The allocation to the UEBMI group (ri = 1) or URRMI (ri = 0) group was used as a criterion variable to indicate differences in the concentration of hospitalization expenses for UEBMI and URRMI. Larger absolute values of the concentration index indicate a more concentrated distribution of costs, whereas values close to 0 indicate a less concentrated distribution of hospitalization expenses. A concentration index below 0 indicates that hospitalization expenses are concentrated in the UEBMI group, whereas above 0 indicates that they are concentrated in the URRMI group.

The significance level for statistical tests was established at *α* = 0.05, with all statistical analyses conducted using Stata 17.0.

## 3 Results

### 3.1 Basic information about the sample

Table 1 presents the distribution of patients in the UEBMI and URRMI groups across disease types after PSM, spanning from 2020 to 2023. Following propensity score matching of the 98,835 pre-processed data entries on an annual basis, a total of 30,300 patients were enrolled in this study, with 13,644 cases of cerebral infarction and 16,656 cases of coronary atherosclerotic heart disease. The URRMI and UEBMI groups were equally represented, accounting for 50% of the total. The data reveal an uneven gender distribution among patients treated for cerebral infarction and coronary atherosclerotic heart disease, with male patients significantly outnumbering female patients, with a male-to-female ratio approaching 7:3. In terms of age distribution, patients aged 60–80 years predominated, accounting for over 60% of the total.

**Table 1:** Basic information of the sample.

Diseases	Variable		URRMI Group		UEBMI group	
			Frequency	Proportion (%)	Frequency	Proportion (%)
CI	Gender	Male	5,018	73.6%	5,029	73.7%
		Female	1804	26.4%	1793	26.3%
	Age	<50	306	4.5%	296	4.3%
		50–60	1,070	15.7%	1,024	15.0%
		60–70	1940	28.4%	1799	26.4%
		70–80	2,524	37.0%	2,555	37.5%
		>80	982	14.4%	1,148	16.8%
CAD	Gender	Male	5,546	66.6%	5,544	66.6%
		Female	2,782	33.4%	2,784	33.4%
	Age	<50	390	4.7%	374	4.5%
		50–60	1,647	19.8%	1,418	17.0%
		60–70	2,409	28.9%	2,186	26.2%
		70–80	2,757	33.1%	2,781	33.4%
		>80	1,125	13.5%	1,569	18.8%

Table 2 displays the hospitalization expenses and length of hospital stay for UEBMI and URRMI treated for cerebral infarction and coronary atherosclerotic heart disease across levels of hospitals. The table’s indicators illustrate the impact of the DIP reform on controlling hospitalization expenses for patients at levels of hospitals.

**Table 2:** Hospitalization expenses and length of hospital stay at all levels of hospitals for UEBMI and URRMI, 2020–2023 (CI, CAD).

Items		Before the reform				After the reform			
		URRMI group		UEBMI group		URRMI group		UEBMI group	
		Average hospitalization expenses	Average length of stay	Average hospitalization expenses	Average length of stay	Average hospitalization expenses	Average length of stay	Average hospitalization expenses	Average length of stay
CI	Primary hospitals	7680.79 ± 4094.76	23.28 ± 19.49	3097.74 ± 1756.63	12.92 ± 13.29	8245.14 ± 3589.33	18.74* ± 8.76	2791.49* ± 1524.70	10.84** ± 8.05
	Secondary hospitals	14046.6 ± 6603.24	17.01 ± 13.93	7322.97 ± 5083.75	15.17 ± 13.85	13122.54*** ± 5477.52	16.15 ± 19.62	7770.63* ± 5151.02	13.59* ± 13.24
	Tertiary hospitals	20017.9 ± 6592.66	14.53 ± 20.48	15969.71 ± 8166.84	15.04 ± 11.90	17528.31*** ± 6060.44	12.09*** ± 7.58	15008.24* ± 7486.27	12.87*** ± 11.46
CAD	Primary hospitals	6568.63 ± 3730.73	16.16 ± 8.89	2759.59 ± 1564.00	11.79 ± 8.3	5981.77* ± 1838.26	14.98 ± 6.45	2562.64 ± 1320.31	9.90** ± 11.12
	Secondary hospitals	11423.29 ± 6836.17	13.71 ± 23.66	6793.88 ± 4987.84	13.98 ± 22.73	10459.92*** ± 5711.02	10.90*** ± 12.24	6987.27 ± 4929.50	10.73*** ± 8.93
	Tertiary hospitals	15388.57 ± 8487.85	8.02 ± 7.89	10895.88 ± 7065.23	12.72 ± 35.17	14130.43*** ± 7047.99	7.86 ± 8.25	10572.51 ± 6777.88	9.18*** ± 19.61

^ns^*p* > 0.05, *p < 0.05, **p < 0.01, ***p < 0.001.

Cross-sectional comparisons indicate that as the hospital level increases, the average hospitalization costs for patients with CI and CAD also rise. A comparative analysis of hospitalization expenses reveals that URRMI consistently exhibits higher average hospitalization costs across all hospital levels when compared to UEBMI. Significant differences exist in the average hospitalization expenses between the two patient groups across various hospital levels. Compared to URRMI, the expense gap between primary and tertiary hospitals is especially notable for UEBMI. Upon observing the length of hospital stay, it was found that the gap in the average length of hospital stay between different levels of hospitals is the largest for URRMI.

The longitudinal comparison revealed that DIP reform significantly reduced the hospitalization expenses of the URRMI group across various diseases, particularly in secondary and tertiary hospitals, with a highly significant *p*-value (*p* < 0.001). For the UEBMI group, the change in the length of hospital stay in the UEBMI group was the most significant (*p* < 0.05), especially in tertiary hospitals. Furthermore, an examination of the *p*-values indicated that the DIP reform had the greatest impact on both hospitalization expenses and length of hospital stay for patients treated for cerebral infarction in tertiary hospitals, with both p-values <0.05.

### 3.2 Trends in the standard deviation of hospitalization expenses

Table 3 shows the SDs of the hospitalization expenses and their changes and double differences for the URRMI and UEBMI groups. The SDs are used to illustrate the changes in the hospitalization expenses of the groups, and the double differences can describe the trend of the differences in the hospitalization expenses between URRMI and UEBMI groups.

**Table 3:** Standard deviation and double-difference value of hospitalization expenses.

Year	URRMI group		UEBMI group		Double-difference value
	SD	Change	SD	Change	
CI					
Before the DIP reform					
2020	7290.62	–	7397.66	–	–
After the DIP reform					
2021	7033.18	−257.44	7815.15	417.49	674.93
2022	5540.97	−1492.21	6632.90	−1182.25	309.96
2023	4852.34	−688.62	5618.11	−1014.78	−326.16
CAD					
Before the DIP reform					
2020	7399.34	–	6035.56	–	–
After the DIP reform					
2021	6594.47	−804.86	6164.55	128.99	933.86
2022	6278.35	−316.12	6342.36	177.80	493.92
2023	6044.13	−234.22	5625.13	−717.22	−483.00

In terms of changes in the standard deviation of hospitalization expenses for patients with cerebral infarction, the standard deviation of hospitalization expense for URRMI declined each year from 2020 to 2023, with the largest decrease observed in 2022, which was 1492.21 less than the previous year, indicating improved cost consistency. The standard deviation of UEBMI rose in the first year of the reform (2021), but subsequently decreased annually thereafter, with a significant reduction. The standard deviation (SD) exhibited the largest change in 2022, decreasing by 1182.25 compared to 2021. When analyzing the expense data for coronary atherosclerotic heart disease patients, we found that the standard deviation of URRMI hospitalization expenses decreased annually, with the most notable drop in 2021, reaching −804.86. In 2020, the standard deviation of hospitalization expenses for UEBMI was 6,035.56. It increased slightly in 2021 and 2022, but the increase was not significant. Overall, the hospitalization expenses still exhibited a decreasing trend. The data suggests that the DIP reform has positively impacted the consistency of hospitalization expenses for URRMI and UEBMI.

In terms of the double-difference value changes, the highest was observed in the first year after the DIP reform (2021) for the double-difference value of hospitalization expenses among URRMI and UEBMI groups treated for cerebral infarction, reaching 674.93. The double-difference value between the expenses of the two groups in 2022 was 309.96, and −326.16 in 2023. Similarly, observing the expense data of patients treated for coronary atherosclerotic heart disease, the double-difference value changed from 933.86 and 493.92 to −483.00. The above indicates that the impact of the DIP reform on the change in hospitalization expenses for the URRMI and UEBMI groups gradually decreased.

### 3.3 Trends in the interquartile range of hospitalization expenses

**Figure 2:**
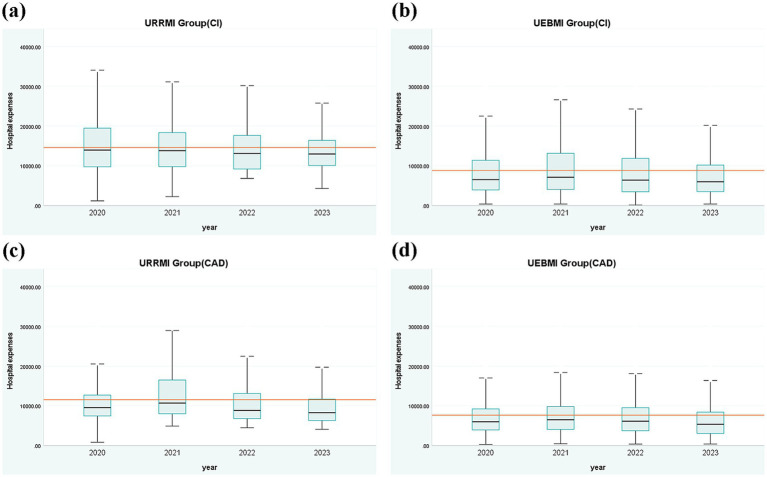
Box plots of hospitalization expenses for different diseases.

Figure 2 presents the distribution of hospitalization expenses for patients with cerebral infarction and coronary atherosclerotic heart disease among URRMI and UEBMI from 2020 to 2023. The figure illustrates the upper and lower bounds of expenses, the median, and the interquartile range (IQR) to reflect the degree of concentration and changes in variation in hospitalization expenses. The red line represents the fixed payment for a particular group of patients in 2020–2023. For patients with cerebral infarction, the IQR values of the URRMI group exhibited a decreasing trend. At the same time, the IQR of the UEBMI initially increased from 2020 to 2021 and then decreased annually thereafter. The median hospitalization expenses and fixed payment amount for the UEBMI group were consistently lower than those of the URRMI group. Furthermore, the difference between the median and the fixed payment amount widened for both groups, with a greater disparity observed in the UEBMI compared to the URRMI. For patients with coronary atherosclerotic heart disease, the IQR value of the URRMI group experienced an initial increase from 2020 to 2021, followed by a yearly decrease. In contrast, the IQR of the UEBMI group showed a non-significant change but demonstrated a general downward trend. The median of the URRMI group increased in the first year after the reform (2021) and then decreased year by year. The median hospitalization expenses and fixed payment amount for the UEBMI group were consistently lower than those of the URRMI group. The difference between median and fixed expenses for both groups was smallest in the first year after the reform (2021) and then gradually increased thereafter. These findings indicate that the DIP reform positively impacted the consistency of hospitalization expenses for both URRMI and UEBMI, with expenses tending to be concentrated.

**Figure 3:**
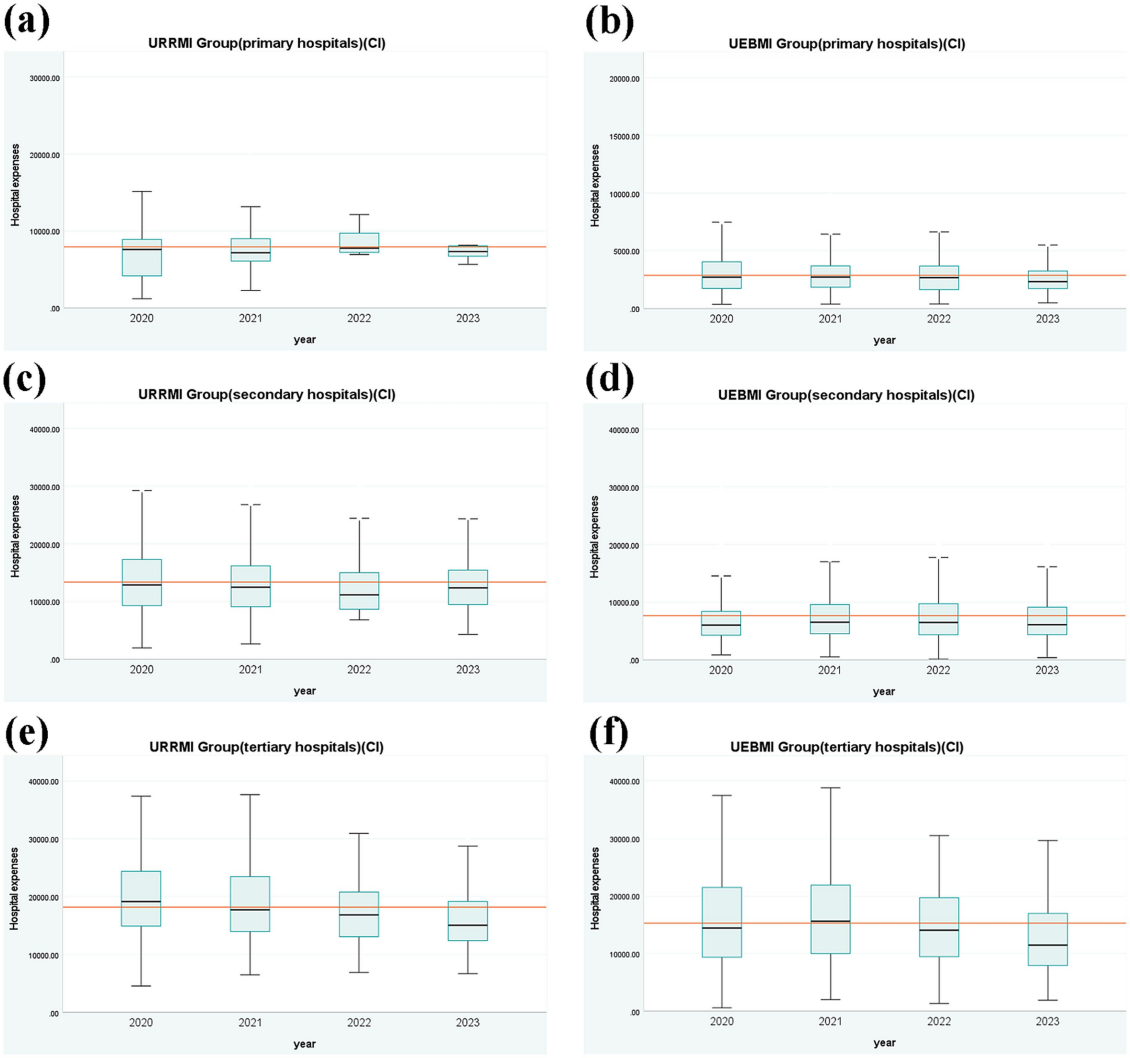
Box plots of hospitalization expenses for different levels of hospitals (CI).

Figure 3 represents the distribution of hospitalization expenses for URRMI and UEBMI treated for cerebral infarction at all levels of hospitals from 2020 to 2023. For primary hospitals, the IQR values of the URRMI group gradually decreased, with the median remaining relatively stable. The IQR of the UEBMI group exhibited a fluctuating downward trend, with a slight increase in 2022 followed by a decrease. For secondary hospitals, the URRMI group’s IQR declined annually, with the median initially decreasing and then increasing in 2023. The IQR value of the UEBMI group initially increased in 2022 and then decreased. For tertiary hospitals, the IQR value and median of the URRMI group exhibited a decreasing trend, with a narrowing gap between the upper and lower limits. The IQR value of the UEBMI group also exhibited a decreasing trend, with the median initially increasing and then decreasing from 2020 to 2021. The comparison of the two groups showed that the average hospitalization expenses and the median of the URRMI group were slightly higher than those of the UEBMI group. Overall, as the hospital level increased, the average hospitalization expenses for both the URRMI and UEBMI groups increased, with the gap between the two groups narrowing and the IQR widening. Generally, patient expenses exhibited decreased variability and became more concentrated.

**Figure 4:**
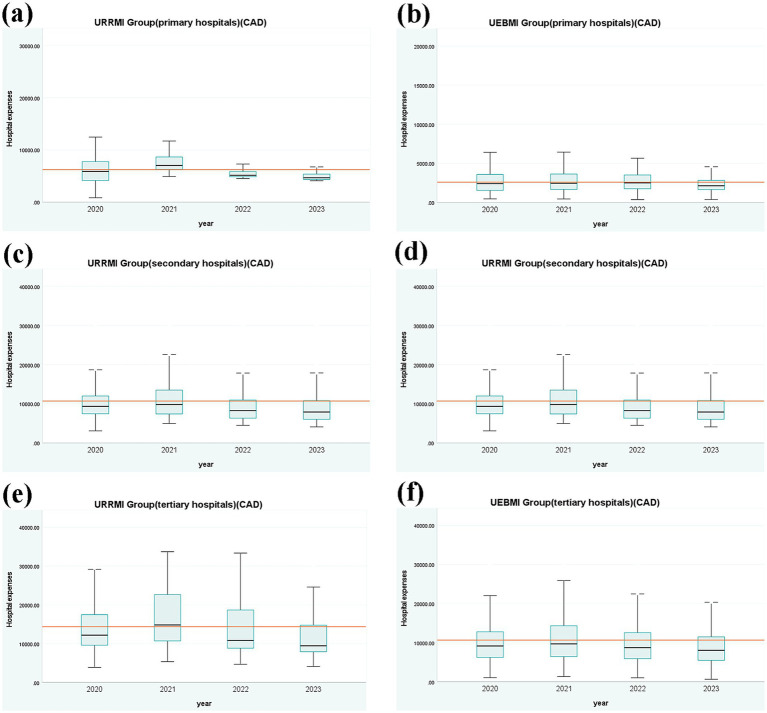
Box plots of Hospitalization expenses for different levels of hospitals (CAD).

Figure 4 presents the distribution of hospitalization expenses for URRMI and UEBMI with coronary atherosclerotic heart diseases treated at various levels of hospitals from 2020 to 2023. For primary hospitals, IQR values in the URRMI group decreased yearly. The median first increased and then decreased in 2021, exceeding the overall average in 2021 and having the largest gap with the overall average hospitalization expenses in 2023. The IQR and median of the UEBMI exhibited a fluctuating downward trend. For secondary hospitals, both the IQR and median of the URRMI group initially increased in 2021 and then decreased. The IQR and median of the UEBMI group showed little variation. For tertiary hospitals, both the IQR values of the URRMI and UEBMI groups initially increased in 2021 and then decreased. The reason for the change in the IQR values and median trend between the URRMI and the UEBMI groups in 2021 may be that under the DIP model, hospitals need to optimize treatment plans to reduce costs while ensuring treatment quality. And since coronary atherosclerotic heart disease is a complex condition, it often necessitates a multitude of medical interventions, including pharmacological therapy and interventional procedures. Hospitals may need to invest more resources and time, which may lead to a temporary increase in expenses. Overall, as the hospital level increased, both the URRMI and UEBMI groups exhibited higher average hospitalization expenses and greater IQR values. Upon comparison, the URRMI group demonstrated slightly higher average and median hospitalization expenses compared to the UEBMI group.

The above analysis indicates that the DIP reform has effectively reduced hospitalization expenses for URRMI and UEBMI treated for cerebral infarction and coronary atherosclerotic heart disease at various levels of hospitals, with expenses tending to become more concentrated.

### 3.4 Concentration index of hospitalization expenses

Table 4 represents the concentration index of hospitalization expenses for patients covered by URRMI and UEBMI from 2020 to 2023. As shown in the figure, the concentration of hospitalization expenses has increased. Despite a brief rise in 2022 and 2023 for cerebral infarction patients, the overall trend remains a fluctuating downward trend, which is statistically significant (*p* < 0.001). For patients with coronary atherosclerotic heart disease, the absolute value of the concentration index rose slightly in the first year of the DIP reform (2021) and 2023. Regarding the difference in expense distribution, the concentration index is less than 0 for both cerebral infarction and coronary atherosclerotic heart disease patients and is statistically significant, indicating that hospitalization expenses are more concentrated in the UEBMI group.

**Table 4:** Concentration index of hospitalization expenses.

Year	CI	*P*	CAD	*P*
Before the DIP reform				
2020	−0.1289404	<0.001	−0.10715998	<0.001
After the DIP reform				
2021	−0.10741875	<0.001	−0.11411076	<0.001
2022	−0.12252005	<0.001	−0.08360314	<0.001
2023	−0.14255038	<0.001	−0.0993642	<0.001

Tables 5–7 present the concentration index of hospitalization expenses for the URRMI and UEBMI in the levels of hospitals from 2020 to 2023. In terms of concentration, the concentration of hospitalization expenses for patients with cerebral infarction and coronary atherosclerotic heart disease treated in primary hospitals has increased. The absolute value of the concentration index for hospitalization expenses among cerebral infarction patients decreased annually after the DIP reform. For patients with coronary atherosclerotic heart disease, the absolute value of the concentration index showed a fluctuating downward trend. In secondary hospitals, the hospitalization expenses for cerebral infarction patients also exhibited a fluctuating downward trend. The absolute value of the concentration index of patients with coronary atherosclerotic heart disease declined year by year after the implementation of the DIP reform. Although there was a brief increase in 2023, this increase was of small magnitude, and the overall downward trend in the absolute value of the concentration index continued. In tertiary hospitals, the absolute value of the concentration index of hospitalization expenses for patients with cerebral infarction declined annually after the DIP reform, with a brief increase in 2023 that was statistically significant (*p* < 0.001). The absolute value of the concentration index for patients with coronary atherosclerotic heart disease increased slightly in 2021, but declined annually thereafter, particularly with a significant decrease in 2022 (*p* < 0.001).

**Table 5:** Concentration index of hospitalization expenses for patients in primary hospitals.

Year	CI	*P*	CAD	*P*
Before the DIP reform				
2020	−0.2140767	<0.001	−0.20481028	<0.001
After the DIP reform				
2021	−0.17725668	<0.001	−0.23065194	<0.001
2022	−0.13948466	<0.001	−0.13557781	<0.001
2023	−0.07634542	<0.001	−0.14058769	<0.001

**Table 6:** Concentration index of hospitalization expenses for patients in secondary hospitals.

Year	CI	*P*	CAD	*P*
Before the DIP reform				
2020	−0.1516358	<0.001	−0.12426722	<0.001
After the DIP reform				
2021	−0.12334739	<0.001	−0.11207911	<0.001
2022	−0.11014197	<0.001	−0.08209652	<0.001
2023	−0.13251721	<0.001	−0.08636472	<0.001

**Table 7:** Concentration index of hospitalization expenses for patients in tertiary hospitals.

Year	CI	*P*	CAD	*P*
Before the DIP reform				
2020	−0.05626191	<0.001	−0.08559945	<0.001
After the DIP reform				
2021	−0.03778337	<0.001	−0.08901698	<0.001
2022	−0.03471399	<0.001	−0.063802	<0.001
2023	−0.05471191	<0.001	−0.05476254	<0.001

On the analysis of differences in expense distribution, the concentration index for patients across the levels of hospitals is statistically significant and less than 0, indicating a greater concentration of hospitalization expenses for UEBMI. Despite variations in how the reform is manifested across different hospital levels, the overall trend remains consistent, and expense consistency has been effectively enhanced.

## 4 Discussion

### 4.1 DIP reforms are effective in controlling expenses

The research findings indicate that the hospitalization expenses of URRMI and UEBMI for the treatment of cerebral infarction and coronary atherosclerotic heart disease exhibit a decreasing trend, with a narrowing gap between the two groups. An analysis of the concentration index reveals that the absolute value of these indices for the hospitalization expenses under the URRMI and UEBMI have exhibited a downward fluctuation, tending towards 0. This trend indicates that the effect of the DIP reform has had a significant effect in controlling hospitalization expenses for the treatment of cerebral infarction and coronary atherosclerotic heart disease across different medical insurance schemes. Hospitalization expenses for patients have become more concentrated after the reform. Previous studies by scholars have also demonstrated that reforms in medical insurance payment methods can effectively influence the concentration level and cost control of hospitalization expenses. For instance, regarding the impact of DIP, the payment based on the diagnosis-intervention packet has a positive effect on the standardization of medical service behaviors and the control of the growth of medical expenses (25). However, the concentration index of hospitalization expenses across different disease categories is less than 0 (statistically significant, *p* < 0.001), which leads to the conclusion that the concentration index of medical costs of UEBMI is always higher than that of URRMI before and after the implementation of DIP. This may be related to the inequality in the economic income levels of URRMI and UEBMI, the relatively lower reimbursement ratio for URRMI, and complex procedures for reimbursement of medical expenses incurred outside the place of registration impede realizing medical insurance benefits. The above analysis indicates that the DIP expenses reform can effectively control the differences in hospitalization expenses between URRMI and UEBMI, but there are still differences in the homogeneity levels of hospitalization expenses among patients with different medical insurance schemes.

### 4.2 Effectiveness of DIP reform on hospitalization expenses at levels of hospitals

This study examined hospitalization expenses for patients under UEBMI and URRMI across different levels of hospitals, analyzing variations to explore the cost-control effects and equity benefits of the DIP reform.

#### 4.2.1 Uneven distribution of hospitalization expenses

Upon comparing the average hospitalization expenses and the length of hospital stay for URRMI and UEBMI treated in all levels of hospitals from 2020 to 2023. It was found that the average hospitalization expenses for patients covered by URRMI are higher than those of UEBMI. Current studies have indicated that the length of hospital stay is an important factor leading to the high expenses of patients (31, 32). Based on the length of hospital stay, the following reasons for the cost differences are speculated. From the perspective of patients’ economic burden, the data indicates that the average length of hospital stay for URRMI in primary and secondary hospitals is significantly longer than that of UEBMI. The main reason for this could be the higher reimbursement of URRMI in primary and secondary hospitals in the area, which makes URRMI more inclined to seek treatment there. On the other hand, due to the high reimbursement limits and relatively low economic burden of illnesses provided by their medical insurance, the UEBMI enjoys certain health benefits, which to some extent stimulates their demand for medical services (33). As a result, they are more dependent on medical services provided by tertiary hospitals. Consequently, the average hospitalization expenses for the URRMI in primary and secondary hospitals are higher than those for the UEBMI. From the demand for medical services, despite the slightly longer hospital stays for UEBMI patients in tertiary hospitals compared to URRMI patients, URRMI patients incur higher hospitalization expenses. Given factors such as the lower reimbursement rates for URRMI in tertiary hospitals compared to the UEBMI and the tendency of rural patients to seek care at higher-level institutions outside their residence only for complex and severe conditions, which also prolongs their length of hospital stay (34). The above analysis indicates that there is still a need to improve the medical insurance system in China and strengthen the integration of URRMI and UEBMI.

In addition, a horizontal comparison of hospitalization expenses at all levels of hospitals reveals that the higher the hospital level, the greater the average expenses for treating cerebral infarction and coronary atherosclerotic heart disease among both the URRMI and the UEBMI. Notably, the disparity in the hospitalization expenses among the levels of hospitals for UEBMI. This is primarily because patients with severe illnesses tend to prefer tertiary hospitals for treatment (35–37), especially the UEBMI who show a more significant preference for medical services provided by tertiary hospitals (14). From a longitudinal perspective, with the in-depth implementation of the medical insurance system reform based on DIP prospective payment in 2021, the hospitalization expenses for URRMI and UEBMI have been decreasing annually across the different levels of hospitals. Meanwhile, the gap in hospitalization expenses between the URRMI and the UEBMI has gradually narrowed. The above analysis indicates that the DIP system reform can effectively control cost growth and reduce the hospitalization expenses gap between URRMI and UEBMI at all levels of hospitals. However, the loophole in the hospitalization expenses gap between primary hospitals and higher-level hospitals still needs to be repaired through certain healthcare policies to promote a balanced diversion of URRMI and UEBMI from the hospitals at all levels and to prevent a siphoning effect in tertiary hospitals (38).

#### 4.2.2 Reduced variance in hospitalization expenses

The data revealed that following the DIP reform, the gap in average hospitalization expenses among hospitals of different levels significantly narrowed for patients with cerebral infarction and coronary atherosclerotic heart disease. Simultaneously, the standard deviation of hospitalization expenses exhibited varying degrees of decrease. By comparing the concentration indices, we found an upward trend in these indices for both disease treatments across hospitals among patients covered by UEBMI and URRMI. This suggests that the DIP reform effectively reduced the disparities in hospitalization expenses among patients with diverse medical insurance schemes in hospitals at various levels. This achievement can be attributed to the payment mechanism based on DIP, which has incentivized hospitals to actively cut costs, enhance service quality and efficiency, promote the rational allocation of health resources, and bolster hospitals’ management capabilities (39, 40). Controlling cost growth and shortening the average length of hospital stay were key foci of the reform (41, 42). Notably, the concentration index of hospitalization expenses for patients in tertiary hospitals approached 0, reflecting a more concentrated distribution of these expenses in such hospitals. Further comparison of the two groups’ concentration indices revealed that all indices across the levels of hospitals were less than 0 (statistically significant, *p* < 0.001), indicating that the concentration of hospitalization expenses for UEBMI was higher than that for URRMI. In summary, the DIP reform has achieved remarkable results in effectively controlling the disparities and improving the consistency of hospitalization expenses between URRMI and UEBMI across hospitals of different levels. However, some results still vary in terms of homogeneity among patients with different medical insurance schemes and levels of hospitals.

## 5 Limitations

This study aims to explore the differences in hospitalization expenses among patients with different medical insurance schemes under the DIP reform. Still, due to the establishment of the China National Healthcare Security Administration in 2018 and the limited years of data collection before the implementation of the DIP reform, the study is unable to obtain medical expense data over a longer time span. Additionally, the database used lacks selectable variables, thus only the changes in total hospitalization expenses are analyzed, without a breakdown of the components of hospitalization expenses. Furthermore, this study statically focuses on the changes in hospitalization expenses for UEBMI and URRMI, neglecting the impact of factors such as out-of-town medical treatment on the variation in hospitalization expenses. These limitations warrant further in-depth consideration and research.

## 6 Conclusion

This study found that DIP reform effectively reduced the variability of patient hospitalization expenses, increased the concentration of hospitalization expenses for both UEBMI and URRMI, and narrowed the cost gap between patients with different insurance schemes. At the same time, this study also analyzes the variability in hospitalization expenses for patients across levels of hospitals. It concludes that the DIP reform can effectively control the differences in hospitalization expenses for URRMI and UEBMI at different levels of hospitals. However, it is worth noting that the DIP reform has varied the results of homogeneity of patients across different medical insurance schemes and the levels of hospitals. Continuous policy adjustments are still needed to optimize the allocation of healthcare resources.

## Data Availability

The data analyzed in this study is subject to the following licenses/restrictions: The data contains private information of patients. Requests to access these datasets should be directed to Meng Xue-Hui, mengxuehui@aliyun.com.
